# Association of Body Mass Index with Depression, Anxiety and Suicide—An Instrumental Variable Analysis of the HUNT Study

**DOI:** 10.1371/journal.pone.0131708

**Published:** 2015-07-13

**Authors:** Johan Håkon Bjørngaard, David Carslake, Tom Ivar Lund Nilsen, Astrid C. E. Linthorst, George Davey Smith, David Gunnell, Pål Richard Romundstad

**Affiliations:** 1 Department of Public Health, Faculty of Medicine, Norwegian University of Science and Technology, 7491, Trondheim, Norway; 2 Forensic Department and Research Centre Bröset St. Olav’s University Hospital Trondheim, Trondheim, Norway; 3 School of Social and Community Medicine, University of Bristol, Bristol, United Kingdom; 4 MRC Integrative Epidemiology Unit at the University of Bristol, Bristol, United Kingdom; 5 Department of Human Movement Science, Norwegian University of Science and Technology, Trondheim, Norway; 6 Neurobiology of Stress and Behaviour Research Group, School of Clinical Sciences, University of Bristol, Bristol, United Kingdom; Charité-Universitätsmedizin Berlin, Campus Benjamin Franklin, GERMANY

## Abstract

**Objective:**

While high body mass index is associated with an increased risk of depression and anxiety, cumulative evidence indicates that it is a protective factor for suicide. The associations from conventional observational studies of body mass index with mental health outcomes are likely to be influenced by reverse causality or confounding by ill-health. In the present study, we investigated the associations between offspring body mass index and parental anxiety, depression and suicide in order to avoid problems with reverse causality and confounding by ill-health.

**Methods:**

We used data from 32,457 mother-offspring and 27,753 father-offspring pairs from the Norwegian HUNT-study. Anxiety and depression were assessed using the Hospital Anxiety and Depression Scale and suicide death from national registers. Associations between offspring and own body mass index and symptoms of anxiety and depression and suicide mortality were estimated using logistic and Cox regression. Causal effect estimates were estimated with a two sample instrument variable approach using offspring body mass index as an instrument for parental body mass index.

**Results:**

Both own and offspring body mass index were positively associated with depression, while the results did not indicate any substantial association between body mass index and anxiety. Although precision was low, suicide mortality was inversely associated with own body mass index and the results from the analysis using offspring body mass index supported these results. Adjusted odds ratios per standard deviation body mass index from the instrumental variable analysis were 1.22 (95% CI: 1.05, 1.43) for depression, 1.10 (95% CI: 0.95, 1.27) for anxiety, and the instrumental variable estimated hazard ratios for suicide was 0.69 (95% CI: 0.30, 1.63).

**Conclusion:**

The present study’s results indicate that suicide mortality is inversely associated with body mass index. We also found support for a positive association between body mass index and depression, but not for anxiety.

## Introduction

Obesity is associated with an increased risk of depression as well as other mental illnesses[1–3]. In contrast, prospective studies from US[4] and Scandinavia [1, 5] have indicated that suicide risk declines as body mass index increases[6]. This is a paradoxical result since depression and other mental illnesses are the strongest risk factors for suicide[7]. Prospective evidence of the link between obesity and anxiety disorders is, however, scarce and mixed[8]. Disentangling the causal nature of these associations is important as obesity, suicide and common mental illness such as anxiety and depression constitute major public health challenges.

The associations from observational studies of body mass index with mental health outcomes are likely to be influenced by reverse causality or confounding by ill-health. The mechanisms linking body mass index and depression are likely to be convoluted, as several studies have indicated that each may induce or worsen the other[9]. High body mass index could influence the risk of depression via biological mechanisms such as inflammation, dysregulation of hormonal stress systems and the risk of developing physical diseases that may have secondary effects on mental health–while being obese could also have negative psychological effects on, for instance, self-image[9]. Depression could also influence the risk of high body mass index via unhealthy lifestyles as mental illness is associated with physical inactivity and unhealthy diets [10–13]. Likewise, medicines prescribed for some mental illnesses might lead to weight gain[14]. The association between obesity and depression could also be confounded by common causes of both being obese and mental illness such as poor socioeconomic position[15].

The causal mechanisms underlying the association between body weight and suicide are not well understood. Different possible explanations have been suggested. High body mass index might influence suicidal risk directly–for instance through decreased impulsive aggressive behaviours possibly due to the influence of body mass index on several hormones and neurotransmitters like testosterone, leptin, and serotonin levels [6, 16–18]. However, associations could also be influenced by pre-existing substance misuse, severe somatic or mental illness that are associated with low body mass index [6, 18].

The use of instrumental variables has previously proven to be a fruitful way to assess the causal nature of potentially confounded associations, or associations driven by reverse causality[19]. In particular, genetic variants (single nucleotide polymorphisms, SNPs) associated with known functional phenotypes have been used in Mendelian randomization studies to assess causality[20, 21]. The results from such studies on obesity and mental health are however, scarce and mixed. For example, results from a UK study using the polymorphism of the FTO gene as an instrumental variable for obesity, provided evidence that obesity is a risk factor for the development of common mental disorders[22]. On the other hand, results from a Danish study using the polymorphisms of the FTO and MC4R genes[23], and from a collaborative Canadian study using the FTO gene polymorphism[24] as instruments for obesity, suggested a possible protective effect of obesity on psychological distress and depression. Finally, the results from a recent cross national study using different polymorphisms as instruments for increased body mass index did not support a causal relationship between obesity and major depression [25]. However, the use of genetic polymorphisms in such studies is often restricted by lack of precision due to weak associations between the variants and the phenotypic traits. This is particularly a problem when studying suicide, since suicide is relatively rare in most populations[26].

In the present study we use an offspring’s body mass index as an instrument for their parent’s body mass index. This has previously been used as an instrument in studies of cause specific mortality[27] and medical costs[28]. Several twin and adoption studies have found a strong genetic component in both adult and childhood body mass index, with heritability estimates ranging from 0.6 to 0.9 in different studies, and that this association is mainly due to common genes rather than shared family environment [29–32]. Further, offspring body mass index is not likely to be a consequence of parents’ mental illness or suicide risk, and hence, reverse causality should be avoided.

In the present study we have assessed the association of body mass index with symptoms of anxiety and depression and suicide death in a large population study, using an offspring’s body mass index as an instrumental variable for own body mass index.

## Methods

### Study population and data linkage

The present study’s results are based partly on a re-analysis of previously published data [1]. The HUNT study is a large population-based health study conducted in Nord-Trøndelag, a rural county in central Norway with a population of about 130,000. At each of three waves (1984–1986, 1995–1997 and 2006–2008), every resident aged 20 years or over was invited to participate. In addition, 13 to 19 year olds were recruited in three waves (the “YoungHUNT” study). The first and third of these coincided with HUNT2 and HUNT3, respectively and the second took place in 2000–2001. As the whole community was invited to participate in the study, many families were represented in the dataset by members from two or more generations. Full details of the study are available on the HUNT website (http://www.ntnu.no/hunt). Briefly, participants in each wave attended a clinic where their height and weight were measured. Body mass index (kg per metre squared) was calculated from these measures. Participants also completed questionnaires, which in HUNT2 and HUNT3 included the Hospital Anxiety and Depression Scale (HADS) [33].

The questionnaire also provided information on subjects' smoking, alcohol use, exercise, education, employment and marital status. Statistics Norway's Death Registry was used to identify all deaths among HUNT participants between the beginning of HUNT1 (1^st^ January 1984) and 31^st^ December 2009. The international classification of diseases (ICD) code accompanying each death was used to identify deaths by suicide (codes E950-959 and E980-E989 in ICD-9; and codes X600-X849, Y100-Y349, Y870 and Y872 in ICD-10). The present study was approved by the Regional Committee for Medical Research Ethics central Norway—2011/1455/REK midt. Each participant and the parents/legal guardians of the participants younger than 16 years old gave their written consent to participate.

### Data preparation

**Fig 1:**
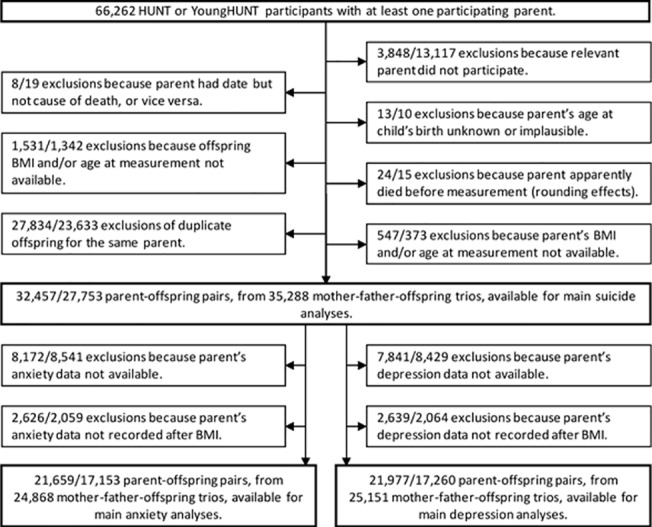
Flow of participants through the study. Numbers are listed as mother-offspring pairs / father-offspring pairs.

We initially extracted data for study members who had at least one participating parent (66,262 offspring, with 62,414 participating mothers and 53,145 participating fathers). Individuals were removed from each analysis if their body mass index data, or the body mass index data of both parents, were missing, and the data were restricted to one randomly chosen offspring per parent. This left 32,457 mother-offspring and 27,753 father-offspring pairs for the main analysis of suicide mortality. The analyses of depression and anxiety additionally required the appropriate parental HADS data, recorded later than the parent's body mass index. This left 21,977 mother-offspring pairs and 17,260 father-offspring pairs in the analysis of depression and 21,659 mother-offspring pairs and 17,153 father-offspring pairs in the analysis of anxiety. The data selection procedure is shown in Fig 1. For those individuals who participated in more than one wave of the study, we used body mass index data from their earliest HUNT (i.e. adult) participation. This minimised the risk of weight loss due to age-related illness, maximised the period at risk for the suicide analysis and maximised the chances of parents' HADS data post-dating their own body mass index data. For offspring only, we used the latest available YoungHUNT body mass index data if no HUNT body mass index data were available. For the analyses of depression and anxiety, we used HADS data from the earliest HUNT wave post-dating the subject's own body mass index measurement. Subjects without HADS data from a later wave than their own body mass index data were not included, but we did not require HADS data to post-date offspring body mass index data (since there is no assertion of causality in this association).

The HADS data consist of seven depression-related and seven anxiety-related questions, each answered on a scale of 0 to 3, giving a total symptom score of 0–21. A value of ≥8 was set to define a case of depression or anxiety [33]. For each outcome, participants were excluded from analysis if the answers to four or more of the seven questions were missing. If 1–3 answers were missing, cases were defined from the non-missing data, which were weighted according to each question's mean contribution to the total score.

Each measure of body mass index was assigned to a quintile among participants of the same sex and similar age, measured in the same HUNT wave. Within these quintiles, we summarized offspring and parents' health-related characteristics and characterised their association with body mass index using linear or logistic regression, as appropriate.

Before all further analyses, body mass index in all participants was adjusted for age, sex and year of measurement by taking residuals from sex-specific full factorial regression models against HUNT wave (categorical, with contemporary HUNT and YoungHUNT combined) and a cubic spline of age. These residuals were then divided by the residual standard deviation of the model to give sex-specific Z-scores of body mass index. Cubic splines used five knots, because the size of the data set permitted a reasonably high precision in the fitting of the relationship. The knots were placed at percentiles of 5, 27.5, 50, 72.5, 95, following the recommendations of Harrell et al. [34].

### Statistical analysis of depression and anxiety

Symptoms of depression and anxiety were analysed separately, using logistic regression models to estimate the odds ratio (OR) for depression/anxiety per sex-specific standard deviation of (i) a subject's own body mass index, and (ii) their offspring's body mass index. All models were adjusted for parental date of birth and age using cubic splines with 3 knots at percentiles of 10, 50, 90. Three knots were chosen to avoid over-adjusting in the analysis of relatively rare binary outcomes, and their positions were those recommended by Harrell et al. [34]. Models were run with and without adjustment for parental education, coded as missing (14% of mothers, 18% of fathers), <10, 10–12, or >12 years and recorded at the same time as the parent's body mass index, as a representation of socioeconomic position. The linearity of associations was assessed by plotting OR by quartiles of adjusted body mass index. Models were run separately for mothers and fathers, but also for both parents together. These combined models were additionally adjusted for parental sex, and used robust standard errors clustered by offspring identity to account for the non-independence of an offspring's two parents. The difference between maternal and paternal OR was tested by adding an interaction between parental sex and the exposure (i.e. own or offspring body mass index) and examining the p-value from a Z-test of the resulting coefficient.

Two-sample instrumental variable IV analysis was used to estimate parental OR for depression/anxiety per sex-specific standard deviation of parental body mass index, with offspring body mass index as an instrument. This method avoids some of the sources of confounding/reverse causality between parental depression/anxiety and body mass index [35, 36]. First, parental body mass index (mothers, fathers and combined parents in turn) was regressed on offspring body mass index, with adjustment as described above for the corresponding logistic regression. Two-sample IV estimates of the OR were made by dividing the natural logarithm of the OR per SD of offspring body mass index (with or without adjustment for education) by the comparably adjusted regression coefficient for parental body mass index against offspring body mass index, and exponentiating. Confidence intervals were calculated using Taylor series expansions [37]. OR calculated by two-sample IV were compared with conventional OR per SD of the parent's own body mass index using 1,000 bootstrap resamples.

### Statistical analysis of suicide mortality

Suicide mortality was analysed similarly to depression and anxiety, but with the use of Cox regression in place of logistic regression. Parental age was used as the time axis, so adjustment for parental age was not necessary. Observations were censored to restrict them to the period when a parent's death would have been recorded and used in the study. Left-censoring was thus applied at the latest of the offspring's date of birth and the parent's body mass index measurement, and right-censoring applied at the earliest of the parent's date of death or emigration, or 31^st^ December 2009 (the end of follow-up). We did not left-censor on the date of the offspring's body mass index measurement, because offspring body mass index was used as an instrument for parental body mass index during life, regardless of whether the parent was alive when the offspring's body mass index was measured.

## Results

### Results—general

The health-related characteristics of parents and their offspring by quintiles of offspring body mass index are presented in Table 1. The parents of offspring with higher body mass index, and the offspring themselves, had higher blood pressure and were less educated and less physically active. The parents of high-body mass index offspring had higher body mass index, were slightly younger at the time of the child's birth, and were also more likely to smoke. Offspring in the lowest quintile of body mass index, and their fathers, were slightly more likely to drink frequently, but this pattern was not repeated in mothers, and there was no clear trend over the other four quintiles. The standard deviation of body mass index was 3.4 kg m^-2^ for women and 4.3 kg m^-2^ for men.

**Table 1:** Characteristics of parents and offspring according to quintiles of offspring body mass index.

	Quintile^1^ of offspring BMI					Linear or logistic regression per SD		
Subject, Measurement	1^st^	2^nd^	3^rd^	4^th^	5^th^	Estimate	95% CI	N
Offspring								
Mean BMI (kg m^-2^)	20.0	22.0	23.4	25.3	29.6	3.84^2^	(3.82–3.86)	35,288
Mean systolic blood pressure (mm Hg)	122.4	123.6	125.0	127.3	131.0	3.60^2^	(3.44–3.75)	34,505
Mean diastolic blood pressure (mm Hg)	72.8	72.8	73.4	74.8	77.4	1.93^2^	(1.81–2.06)	34,508
Proportion ever smoked (%)	40.8	37.3	37.3	38.6	39.7	1.02^3^	(1.00–1.04)	32,361
Proportion drinking > = 5 times fortnightly (%)	4.7	3.8	3.7	4.0	3.6	0.90^3^	(0.84–0.97)	22,754
Proportion educated > = 10 years (%)	73.4	76.9	76.2	72.8	69.4	0.94^3^	(0.90–0.97)	18,431
Proportion physically active (%)	89.5	92.0	93.0	91.7	89.3	0.96^3^	(0.91–1.01)	20,056
Mothers								
Mean BMI (kg m^-2^)	24.1	24.6	25.1	25.6	26.8	0.98^2^	(0.93–1.03)	33,000
Mean systolic blood pressure (mm Hg)	133.8	133.1	133.7	134.9	135.5	0.55^2^	(0.27–0.82)	32,823
Mean diastolic blood pressure (mm Hg)	81.2	81.1	81.3	81.9	82.4	0.47^2^	(0.33–0.60)	32,816
Mean age at child's birth (years)	27.5	27.5	27.4	27.2	27.2	-0.19^2^	(-0.26–-0.13)	33,172
Proportion ever smoked (%)	44.4	45.8	47.6	48.9	52.3	1.16^3^	(1.13–1.19)	28,382
Proportion drinking > = 5 times fortnightly (%)	2.7	2.9	2.9	3.0	2.6	1.00^3^	(0.93–1.07)	27,761
Proportion educated > = 10 years (%)	44.9	46.0	45.3	43.0	40.9	0.93^3^	(0.91–0.95)	27,049
Proportion physically active (%)	85.9	86.1	86.3	84.5	84.7	0.95^3^	(0.91–0.98)	23,490
Fathers								
Mean BMI (kg m^-2^)	24.6	25.1	25.4	25.8	26.6	0.73^2^	(0.69–0.76)	28,133
Mean systolic blood pressure (mm Hg)	138.8	138.2	138.8	138.9	140.5	0.46^2^	(0.22–0.70)	27,993
Mean diastolic blood pressure (mm Hg)	84.7	85.0	85.2	85.2	85.9	0.48^2^	(0.34–0.61)	27,988
Mean age at child's birth (years)	30.6	30.5	30.4	30.3	30.5	-0.09^2^	(-0.17–-0.02)	28,381
Proportion ever smoked (%)	63.1	61.6	61.7	64.3	66.5	1.09^3^	(1.06–1.12)	24,217
Proportion drinking > = 5 times fortnightly (%)	8.9	7.9	7.9	8.0	7.6	0.96^3^	(0.91–1.01)	23,652
Proportion educated > = 10 years (%)	51.9	53.3	51.1	48.6	45.3	0.89^3^	(0.87–0.92)	22,863
Proportion physically active (%)	86.1	86.7	85.7	84.2	83.3	0.90^3^	(0.87–0.94)	19,830

^1^Quintiles were calculated among participants of the same sex and similar age, measured at the same survey occasion.

^2^Linear regression per standard deviation (4.33 kg m^-2^ in women and 3.39 kg m^-2^ in men) of offspring BMI, adjusted for age, sex and survey occasion; regression coefficients are presented.

^3^Logistic regression per standard deviation (4.33 kg m^-2^ in women and 3.39 kg m^-2^ in men) of offspring BMI, adjusted for age, sex and survey occasion; odds ratios (exponentiated coefficients) are presented.

For the analysis of depression, there were 2,640 maternal and 2,159 paternal cases of depression among the sample of 21,977 mothers and 17,260 fathers. For the analysis of anxiety, there were 3,902 maternal and 1,899 paternal cases of anxiety among the sample of 21,659 mothers and 17,153 fathers. For the analysis of suicide, there were 34 maternal and 101 paternal suicides among the sample of 32,457 mothers and 27,761 fathers.

### Association between offspring and parental body mass index

Regression coefficients for parental body mass index against offspring body mass index, used in the calculation of two-sample IV estimates, are shown in Table 2. Values were calculated separately for each outcome because slightly different subsets of the data were used. In the data subset for the analysis of depression, for each standard deviation increase in combined parental body mass index, there was a 0.21 (95% CI: 0.20, 0.22) fully adjusted standard deviation increase in the offspring body mass index. Similar values were obtained regardless of the parent in question, the adjustment used, and the data subset.

**Table 2:** Regression coefficients between parental and offspring body mass index within the data set used for each outcome.

		Coefficient (95% CI) for Outcome:		
Parent	Adjustment	Depression	Anxiety	Suicide
Mothers	Minimal	0.22 (0.21, 0.23)	0.22 (0.21, 0.23)	0.24 (0.23, 0.25)
	Full	0.22 (0.20, 0.23)	0.22 (0.20, 0.23)	0.24 (0.23, 0.25)
N		21,977	21,659	32,457
Fathers	Minimal	0.20 (0.19, 0.22)	0.20 (0.19, 0.21)	0.22 (0.21, 0.23)
	Full	0.20 (0.18, 0.21)	0.20 (0.18, 0.21)	0.22 (0.21, 0.23)
N		17,260	17,153	27,753
Combined	Minimal	0.21 (0.20, 0.22)	0.21 (0.20, 0.22)	0.23 (0.22, 0.24)
	Full	0.21 (0.20, 0.22)	0.21 (0.20, 0.22)	0.23 (0.22, 0.24)
N		39,237	38,812	60,210

Note: Body mass index was pre-adjusted for age, sex and HUNT wave and standardised by the sex-specific residual standard deviation. All models were adjusted for parental age and date of birth and full adjustment included additional adjustment for parental education. Models for combined parents were additionally adjusted for parental sex and used robust standard errors clustered by offspring identity.

### Body mass index and depression symptoms

**Fig 2:**
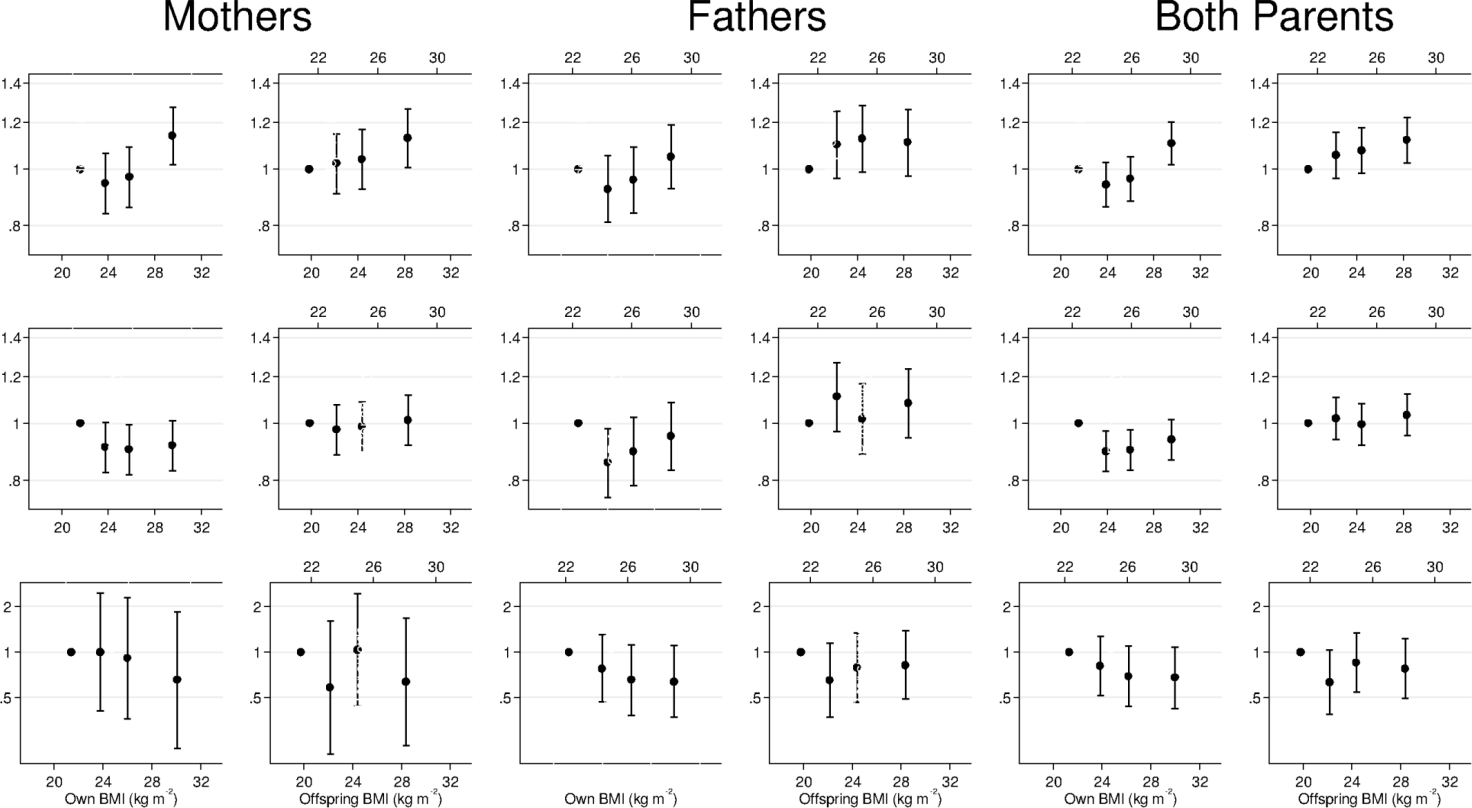
Odds ratios (OR) for depression and anxiety, and hazard ratios (HR) for suicide mortality, by quartiles of own and offspring body mass index relative to the smallest quartile. Body mass index was pre-adjusted for age and HUNT wave and converted to sex-specific Z scores before classification into quartiles. It is plotted as the mean body mass index for each quartile, converted back to kg m^-2^ for men (upper X-axis) and women (lower X-axis). All models were adjusted for parental age, date of birth and education. Note the different scale on the y-axis for suicide.

Mothers and fathers showed similar positive associations between body mass index and depression (Table 3, Fig 2). Full adjustment attenuated the odds ratios a little for own body mass index and for offspring body mass index. The wide confidence intervals made it difficult to infer the exact shape of the association, but there was no evidence of non-linearity (Fig 2). OR from two-sample IV analyses were rather higher than those from conventional analyses using subjects' own body mass index, but with wide confidence intervals, and bootstrap comparisons provided only some evidence for a difference in odds ratios between the analyses using a subject's own body mass index and the IV analyses (with minimal adjustment: p = 0.06 for mothers and p = 0.09 for fathers and with full adjustment: p = 0.13 for mothers and p = 0.27 for fathers).

**Table 3:** Odds ratio for depression (HADD> = 8) per sex-specific SD of body mass index.

		Mothers		Fathers		Comparison	Combined	
Adjustment	Exposure	OR	95% CI	OR	95% CI	p	OR	95% CI
Minimal	Own BMI	1.08	1.03, 1.13	1.07	1.01, 1.12	0.71	1.08	1.04, 1.11
Minimal	Offspring BMI	1.06	1.02, 1.11	1.05	1.00, 1.10	0.83	1.06	1.02, 1.09
Minimal	IV	1.31	1.08, 1.59	1.29	1.02, 1.63		1.30	1.12, 1.52
Full	Own BMI	1.06	1.02, 1.11	1.05	0.99, 1.10	0.66	1.06	1.02, 1.09
Full	Offspring BMI	1.05	1.00, 1.09	1.04	0.99, 1.09	0.69	1.04	1.01, 1.08
Full	IV	1.24	1.02, 1.51	1.19	0.94, 1.52		1.22	1.05, 1.43

Note: Body mass index was pre-adjusted for age, sex and HUNT wave and standardised by the sex-specific residual standard deviation. All models were adjusted for parental age and date of birth and full adjustment included additional adjustment for parental education. Models for combined parents were additionally adjusted for parental sex and used robust standard errors clustered by offspring identity. Maternal and paternal odds ratios were compared by adding an interaction between parental sex and the exposure to the combined model and reporting the p-value from a Z-test of its coefficient. There were 2,640 cases of depression among 21,977 mothers and 2,159 cases of depression among 17,260 fathers.

### Body mass index and Anxiety symptoms

There was no evidence of an association between anxiety and a person's own body mass index (Table 4, Fig 2). When an offspring's body mass index was used as an instrument for their parents' body mass index, there was some evidence of a positive association, but this was somewhat attenuated by full adjustment, and 95% confidence intervals before and after adjustment included the null. We found only weak evidence for a difference in odds ratios between the analyses using a subject's own body mass index and the IV analyses with minimal adjustment (bootstrap comparisons; p = 0.09 for mothers and p = 0.14 for fathers),or with full adjustment (bootstrap comparisons; p = 0.21 for mothers and p = 0.25 for fathers). Odds ratios were similar for mothers and fathers.

**Table 4:** Odds ratio for anxiety (HADA> = 8) per sex-specific SD of body mass index.

		Mothers		Fathers		Comparison	Combined	
Adjustment	Exposure	OR	95% CI	OR	95% CI	p	OR	95% CI
Minimal	Own BMI	0.98	0.94, 1.02	1.02	0.96, 1.07	0.39	0.99	0.96, 1.02
Minimal	Offspring BMI	1.03	0.99, 1.06	1.04	0.99, 1.09	0.51	1.03	1.00, 1.06
Minimal	IV	1.12	0.95, 1.32	1.22	0.96, 1.56		1.15	1.00, 1.33
Full^1^	Own BMI	0.96	0.93, 1.00	1.00	0.95, 1.06	0.42	0.98	0.94, 1.01
Full^1^	Offspring BMI	1.02	0.98, 1.05	1.03	0.98, 1.08	0.59	1.02	0.99, 1.05
Full^1^	IV	1.07	0.91, 1.27	1.16	0.90, 1.49		1.10	0.95, 1.27

Note: Body mass index was pre-adjusted for age, sex and HUNT wave and standardised by the sex-specific residual standard deviation. All models were adjusted for parental age and date of birth and full adjustment included additional adjustment for parental education. Models for combined parents were additionally adjusted for parental sex and used robust standard errors clustered by offspring identity. Maternal and paternal odds ratios were compared by adding an interaction between parental sex and the exposure to the combined model and reporting the p-value from a Z-test of its coefficient. There were 3,902 cases of anxiety among 21,659 mothers and 1,899 cases of anxiety among 17,153 fathers.

### Body mass index and suicide

Suicide mortality was inversely associated with body mass index, whether a subject's own body mass index, their offspring's, or an instrumental variables approach was used (Table 5, Fig 2). However, the wide confidence intervals around these hazard ratios, particularly for women, mean that this result should be interpreted cautiously. We found no statistical evidence that hazard ratios for suicide mortality differed between mothers and fathers (Table 4), or that hazard ratios calculated conventionally from own body mass index differed from those calculated by IV (fully adjusted analysis of 1000 bootstrap resamples comparing IV hazard ratios with conventional ones; p = 0.80 in mothers and 0.85 in fathers). Adjustment did not substantially alter the hazard ratios.

**Table 5:** Hazard ratios for suicide mortality per sex-specific SD of body mass index.

		Mothers		Fathers		Comparison	Combined	
Adjustment	Exposure	HR	95% CI	HR	95% CI	p	HR	95% CI
Minimal	Own BMI	0.81	0.54, 1.22	0.82	0.65, 1.03	0.923	0.82	0.67, 1.00
Minimal	Offspring BMI	0.91	0.63, 1.31	0.94	0.76, 1.16	0.952	0.93	0.76, 1.13
Minimal	IV	0.67	0.15, 3.05	0.74	0.28, 1.93		0.73	0.31, 1.70
Full	Own BMI	0.79	0.53, 1.19	0.81	0.65, 1.03	0.925	0.81	0.67, 0.99
Full	Offspring BMI	0.89	0.62, 1.28	0.93	0.75, 1.15	0.972	0.92	0.76, 1.12
Full	IV	0.61	0.13, 2.83	0.71	0.27, 1.88		0.69	0.30, 1.63

Note: Body mass index was pre-adjusted for age, sex and HUNT wave and standardised by the sex-specific residual standard deviation. All models were adjusted for parental date of birth and full adjustment included additional adjustment for parental education. Parental age was used as the time axis. Models for combined parents were additionally adjusted for parental sex and used robust standard errors clustered by offspring identity. Maternal and paternal hazard ratios were compared by adding an interaction between parental sex and the exposure to the combined model and reporting the p-value from a Z-test of its coefficient. There were 34 suicide deaths among 32,457 mothers and 101 suicide deaths among 27,753 fathers.

## Discussion

In this large population study, we studied the associations between body mass index and depression, anxiety and suicide, using an offspring’s body mass index as an instrumental variable for parental body mass index. Our results indicated that body mass index and depression were positively associated, while the results did not indicate any substantial association between body mass index and anxiety. The results indicated that suicide mortality was inversely associated with body mass index, but the wide confidence intervals around the estimates made by instrumental variable methods suggest that this result should be interpreted with care and leaves chance variation as an alternative explanation. These findings are in keeping with our earlier analysis of HUNT participants' own body mass index in relation to depression, anxiety and suicide[1]. Effect estimates calculated by two-sample instrumental variable methods, particularly those for depression, were further from the null than the equivalent figures calculated by conventional methods using the parent's own body mass index. This suggests that conventional analyses might underestimate the strength of the causal effect of body mass index on the outcomes, due to some form of confounding associating them in the opposite direction although chance variation could also be an alternative explanation.

### Strengths and limitations

Our study was based on data from a large population based study, with clinical measures of the weight and height of both parents and their children. Despite the large sample size, the low incidence of suicide (135 suicides over the follow-up period) left us with limited power to investigate associations of offspring body mass index with suicide risk.

Information on suicide was collected from the National Cause of Death Registry where the positive predictive value of suicide is likely to be high, although suicide might be underreported. Possible underreporting of suicide is however, not likely to bias our results given the prospective approach of the study. We used validated assessments of anxiety and depression[33], and although not feasible in a study of this size, a structured psychiatric diagnostic interview would have given more reliable diagnostic information than the fourteen-item HADS questionnaire. Particularly, our ability to differentiate between anxiety and depression symptoms could be limited given the considerable overlap between symptoms of anxiety and depression[38]. Given the objective measurement of body mass index and the instrumental variable approach, the risk of any differential misclassification should be avoided. However, in the case of non-differential misclassification of anxiety and depression symptoms, we would expect an attenuation of the associations. Hence, the lack of an association between body mass index and anxiety symptoms could also be influenced by measurement error.

Our study included only families with information from mother-offspring, father-offspring or both parents-offspring pairs. Hence, we cannot rule out possible selection effects from excluded participants without participating parents or offspring.

### Instrumental variable assumptions

By using an instrument variable approach, we could arguably provide better estimates of the body mass index-depression,-anxiety and-suicide associations than we could have done using parental body mass index only. Nevertheless, instrumental variable analyses are based on the strength of the association between the instrument and the exposure, and on several key assumptions[19]. There was a strong association between offspring and parental body mass index in our data, but the essential assumption that the instrument should only influence the outcome through the exposure of interest is difficult to evaluate [19]. There is evidence to suggest that the association between parental and offspring body mass index can be largely attributed to common genes and not shared environment [29–32]. The same pattern has been suggested for mental illnesses for which shared family environment seems to play a minor etiologic role in the development of depression[39]. Hence, any association between offspring and parental mental illness would not likely be caused by common environmental features of the family. Furthermore, high offspring body mass index is not likely to be caused by parental ill health or parental poor mental health. Hence, the typical problem with reverse causality and confounding by ill-health in observational studies of consequences of obesity should be avoided in our study using a first degree relative’s body mass index as an instrument for own body mass index. Nevertheless, offspring body mass index might to some extent be related to shared confounding at the family level, and in the present study we had information from a range of possible confounding variables that could have influenced the observed associations in our analyses.

### Comparison with other studies

To our knowledge, this is the first study to use offspring body mass index as an instrument for parental body mass index to study the association of body mass index with depression, anxiety and suicide. The positive association between offspring body mass index and parental depression is in line with former evidence linking obesity to depression[1, 9, 22], although results in the opposite direction have been reported[23, 24]. A recent review indicated positive cross sectional associations between obesity and anxiety disorders and symptoms, while prospective evidence for an effect of obesity on anxiety disorders is scarce and mixed[8]. We did not find substantial evidence of an association between body mass index and anxiety symptoms–a result that might indicate that body mass index is not a cause of anxiety. However, since there is a considerable overlap between symptoms of anxiety and depression, one should be cautious interpreting separate effects of either[38]. Our results give further support to a protective link between high body mass index and suicide risk, a result which has been reported in several other studies[6], although not in a recent study from Taiwan [40] and a study from UK[41]. Further, although there is some evidence that symptoms of depression decrease after bariatric surgery[42], risk of suicide may increase [43]–but the latter observation is based on a relatively small number of events.

### Possible mechanisms

The paradoxical finding that high body mass index is associated with an increased risk of depression but seems to be a protective factor for suicide, was not altered using our analytical approach, although the precision was low for the body mass index-suicide association. Several possible mechanisms have been suggested for the association between obesity and suicide[44–47]. For instance high body mass index might decrease impulsive suicide attempts via its association with several hormones and neurotransmitters [6, 16–18]. However, a Swedish study of attempted suicide found limited evidence that associations differed according to method of self-harm[5]. Confounding by pre-existing substance misuse or severe somatic or mental illness associated with low body mass index is another possible explanation [6, 18]. A large population-based US study investigated the associations between body mass index and several potential risk factors for suicide, but did not find that these risk factors were consistently inversely associated with greater body mass index[45]. Finally, most studies have investigated associations between adult body mass index and suicide risk. Hence, at the start of follow up, mental health status and also treatment might themselves have influenced body weight. A major advantage of the present study is that reverse causality and confounding by ill-health should be avoided since high offspring body mass index is not likely to be caused by poor parental mental health. Hence, although we do not have information on the possible mediating factors, our study indicates that body mass index influences suicidal risk via other causal pathways than mental illness.

Although depression, anxiety and suicide are associated with body mass index and share common risk factors, their underlying neurobiology is likely to be different. This notion is underscored by the diversity of psychiatric illnesses which may culminate in suicide and include depression, bipolar disorder, schizophrenia, PTSD and substance abuse. A large number of studies have shown an important role for serotonin [48] and glucocorticoid hormones[49] in the aetiology of depression. There are further reports suggesting that these systems are malfunctioning also in suicidal behaviour and suicide completion[50]. In this respect it is of interest to note that both body weight control and food intake, and consequently body mass index, are regulated by serotonin [51] and glucocorticoids [52] at the level of the brain. Low levels of brain serotonin result in hyperphagia and an increase in food intake. It is therefore of interest that body mass index seems to be negatively associated with the serotonin transporter binding (SERT) in several parts of the brain including the midbrain[53]. Thus a low body mass index is associated with higher SERT levels in the brain which may result in decreased extracellular levels of serotonin, which could be interpreted as a compensatory drive to stimulate food intake.

Alterations in glucocorticoid hormone physiology in individuals with symptoms of depression and/or anxiety and in suicide victims may involve various levels of the hypothalamic-pituitary-adrenal (HPA) axis. Elevated plasma concentrations of cortisol and abnormal neuroendocrine challenge tests point to a hyperactive HPA axis in depressed patients[49]. Cortisol non-suppression in the Dexamethasone Suppression Test (DST) has been linked to an increased risk of suicide attempts and suicide completion[54]. However, whether changes in HPA axis activity are involved in the negative relationship between body mass index and suicide is presently difficult to confirm.

Currently it is impossible to draw a conclusive and comprehensive picture of the neurobiological mechanisms linking body mass index with depression and suicide completion. However, it is most likely that the exact make-up of changes in serotonergic neurotransmission and glucocorticoid hormone physiology, together with changes in other systems such as GABAergic neurotransmission and also leptin[55], will determine both body mass index and psychopathology, including affective disturbances and suicide.

### Conclusion

Separately and together, anxiety, depression, suicide and obesity constitute substantial challenges on the global burden of disease. Our results give support to a positive association between body mass index and depression, but not anxiety–while the risk of suicide on the other hand, seems to be reduced with increased body mass index. As obesity is an increasingly prevalent disease[56, 57], clinicians should take into account possible depression symptoms in clinical settings with obese patients. Furthermore, the present study’s results indicate that the body mass index–suicide association is not mediated by depression. This calls for further studies investigating the biologic mechanisms underlying suicide. Furthermore, the underlying mechanisms in the associations between obesity and risk of mental illness and suicide are complex and intricate, involving different bio-psycho-social causal pathways. Improved understanding of these causal mechanisms could lead to better personalized medicine for individuals with obesity as well as mental problems like depression.
